# The application of multiplex fluorimetric sensor for the analysis of flavonoids content in the medicinal herbs family *Asteraceae, Lamiaceae, Rosaceae*

**DOI:** 10.1186/0717-6287-48-5

**Published:** 2015-01-16

**Authors:** Oksana Sytar, Klaudia Bruckova, Elena Hunkova, Marek Zivcak, Kiessoun Konate, Marian Brestic

**Affiliations:** Plant Physiology and Ecology Department, Institute of Biology, Taras Shevchenko National University of Kyiv, Volodymyrskya str., 64, Kyiv, 01033 Ukraine; Department of Plant Physiology, Slovak University of Agriculture in Nitra, A.Hlinku 2, Nitra, Slovakia; Laboratory of Biochemistry and Applied Chemistry, University of Ouagadougou, 09 P.O. Box 848, Ouagadougou 09, Burkina Faso

**Keywords:** Non-destructive measurement, Flavonoids, Multiplex fluorimetric sensor, *Asteraceae*, *Lamiaceae*, *Rosaceae*

## Abstract

**Background:**

The aim of our research work was to quantify total flavonoid contents in the leaves of 13 plant species family *Asteraceae*, 8 representatives of family *Lamiaceae* and 9 plant species belonging to family *Rosaceae*, using the multiplex fluorimetric sensor. Fluorescence was measured using optical fluorescence apparatus Multiplex(R) 3 (Force-A, France) for non-destructive flavonoids estimation. The content of total flavonoids was estimated by FLAV index (expressed in relative units), that is deduced from flavonoids UV absorbing properties.

**Results:**

Among observed plant species, the highest amount of total flavonoids has been found in leaves of *Helianthus multiflorus* (1.65 RU) and *Echinops ritro* (1.27 RU), Rudbeckia fulgida (1.13 RU) belonging to the family *Asteraceae*. Lowest flavonoid content has been observed in the leaves of marigold (*Calendula officinalis*) (0.14 RU) also belonging to family *Asteraceae*. The highest content of flavonoids among experimental plants of family *Rosaceae* has been estimated in the leaves of *Rosa canina* (1.18 RU) and among plant species of family *Lamiaceae* in the leaves of *Coleus blumei* (0.90 RU).

**Conclusions:**

This research work was done as pre-screening of flavonoids content in the leaves of plant species belonging to family *Asteraceae, Lamiaceae* and *Rosaceae*. Results indicated that statistically significant differences (P > 0.05) in flavonoids content were observed not only between families, but also among individual plant species within one family.

## Background

Research over decades has sequentially and convincingly revealed considerable biological effects reported to be exerted by herbs and their active bio-ingredients. Increasingly it is being realized that herbs and their active ingredients have wide ranging implications in many domains, including medicine, nutrition, flavoring, beverages, dyeing, repellents, fragrances, cosmetics [1–3]. Many species have been recognized to have medicinal properties and beneficial impact on health, e.g. antioxidant activity, digestive stimulation action, antiinflammatory, antimicrobial, hypolipidemic, antimutagenic effects and anticarcinogenic potential [4]. Crude extracts of herbs and spices, and other plant materials rich in phenolics are of increasing interest in the food industry because they retard lipid peroxidation and thereby improve the quality and nutritional value of food. Rapidly accumulating experimentally verified data is deepening our understanding about considerable activity of flavonoids against cancer cells as evidenced by cell culture studies and flavonoids induced tumor regression in xenografted mice. Flavonoids are also reported to induce apoptosis in TRAIL resistant cancer cells. Confluence of information also emphasized on flavonoids mediated inhibitory effects on oxidative stress induced cellular damage.

The information on the content of some important secondary metabolites in the particular medicinal herbs of family *Asteracea are well known. Echinacea purpurea* L. is one of the most important medicinal herbs and it is a species of *Asteraceae* natively perennial grown in North America, which is used pharmacologically and for aesthetic enjoyment. In 2005, *Echinacea* products ranked among the top botanical supplements sold in the United States [5]. Varieties of *Echinacea purpurea* all contain similar main ingredients including caffeic acid derivatives, alkamides, flavonoids, essential oils, and polyacetylenes, and medicinal activities of which are yet to be exactly identified with corresponding diseases [6]. Its known that *Echinacea purpurea* is well known and widely used herb so would be interesting also to make screening of different representatives of family *Asteraceae*, including *Echinacea purpurea*, regarding flavonoids content and to compare flavonoids content among different representatives. For example, *Tagetes patula* Linn. (*Asteraceae*) is known mostly as a source of essential oil, limonene, caryophyllene from leaves and roots [7]. Representatives of *Helianthus* sp. (*Asteraceae*) are known as a source of provitamin A in the leaves or oil from seeds [8], but the content of flavonoids in the leaves of different representatives is still unknown. The medicinal herb *Calendula officinalis* leaves and flowering top has been used for spasmolytic, inflamed wounds, analgesic effects [8]. Nowadays is available information about antioxidant activity, phenolics and flavonoids content of *Calendula officinalis* flowers extract, but still missing information regarding flavonoid content in the leaves. Summarizing all, a content of flavonoids in the leaves and herbals of common medicinal plants of family *Asteraceae* is still not completely known and the screening information is needed.

Numerous members of *Lamiaceae* family have traditional and medicinal uses and have been used in folk medicine for many years. Most of genera of the *Lamiaceae* are rich sources of terpenoids and they also contain a considerable amount of various iridoid glycosides, flavonoids, and phenolic acids such as rosmarinic acid and other phenolic compounds [9]. 80% of the species of family *Laminaceae* are used for medical purposes. *Lamiaceae* species are mainly used for ailments related to the digestive system, especially flatulence and dyspepsia. These plants are also used as a reconstituent and for the treatment of infections [9]. *Lamiaceae* herbs were also shown to have higher amounts of phenolic compounds and antioxidants compared with herbs belonging to the representatives of other families (*Mentha pulegium*, *Salvia nemorosa*, *Ocimum basilicum*, *Polygonum aviculare* (*Polygonaceae*), *Hibiscus trionum* (*Malvaceae*), *Tanacetum balsamita* (*Asteraceae*), *Cichorium intybus* (*Asteraceae*) and *Borago officinalis* (*Boraginaceae*) [10]. Among *Lamiaceae* family, there are some well known medicinal herbs as lavandula (*Lavandula angustifolia*) and sage (*Salvia officinalis*). The content of total flavonoids and antioxidative capacities in the dried plant material of these medicinal herbs with using wet chemical analyses have been studied well [11]. But information about the content of flavonoids in the fresh leaves is not available as information about the content of some antioxidant in the different plant parts (leaves, herbs etc.).

Flower extracts of *Eriobotrya japonica* (representative of family *Rosaceae*) have been showed very high antioxidant capacity, and it is a potential source of natural antioxidant [12]. Ursolic acid, oleanolic acid, maslinic acid, malic acid, amygdalin, saponins, hyperin and catechin were identified in this plant [13, 14], and some of them could be antioxidant components. The contents of flavonoids and phenolics in the flowers at different developmental stages and in the various flower tissues clearly differed, with the highest flavonoids and phenolics content in flowers of stage 3 (flower fully open) and petal, respectively [15]. The total phenolics and flavonoids contents of fruit *Rosa canina* extracts, another representatives of family *Rosacea*, indicating that extracts containing these compounds are the major contributors of antioxidant properties [16]. The presence of flavonoids and also their concentration in the many representatives of *Rosaceae* family in the leaves also still unknown well.

Traditionally, the measurement of content of secondary metabolites is investigated by using wet chemical analyses. As a rule, these analyses are costly and very laborious. Thus, in the last years efforts were made to identify non-destructive techniques for *in vivo* monitoring of secondary metabolites accumulation in plants. Using the screening techniques [17], flavonol and anthocyanins content in fruits and leaves can be estimated by chlorophyll fluorescence excitation [18–20]. In this case, non-destructive measurement of flavonoids using the device Multiplex ® 3 (Force - A, France) can be actual and useful for sustainable management of medicinal plants. In this non-destructive measurement of flavonoids, as an important point is absence of plant damage compared to the using wet chemical analysis, rapidity and no costs of chemicals. The fluorimetric sensor represents a rapid and non-invasive tool to: (1) monitor the flavonol accumulation in plant material and to assess its quality concerning the healthy anti-oxidant properties; (2) evaluate the effect of environmental and agronomical factors related to the plant material quality; and (3) select plant parts (leaves and herbs) with the highest flavonoids content [21]. It was estimated that the fluorescence-based indices as decadic logarithm of the red to ultraviolet excitation ratio of far-red chlorophyll fluorescence (FLAV), decadic logarithm of the red to green excitation ratio of far-red chlorophyll fluorescence (ANTH RG) and also ultraviolet excitation ratio of blue and far-red chlorophyll fluorescence (BFRR UV) enable the monitoring of flavonoid and centelloside concentrations in plant leaves of *Centella asiatica* L. The fluorescence-based flavonol (FLAV) and anthocyanin (ANTH RG) indices correlated fairly with flavonoid and especially with anthocyanin concentrations [22].

The aim of the study is to examine *in vivo* the accumulation of flavonoids in the leaves of different species of medicinal herbs, belonging to the family *Rosaceae*, *Asteraceae*, *Laminaceae* by means of fluorescence-based non-destructive measurements, using products of the secondary metabolism as reference.

## Result and discussion

**Figure 1:**
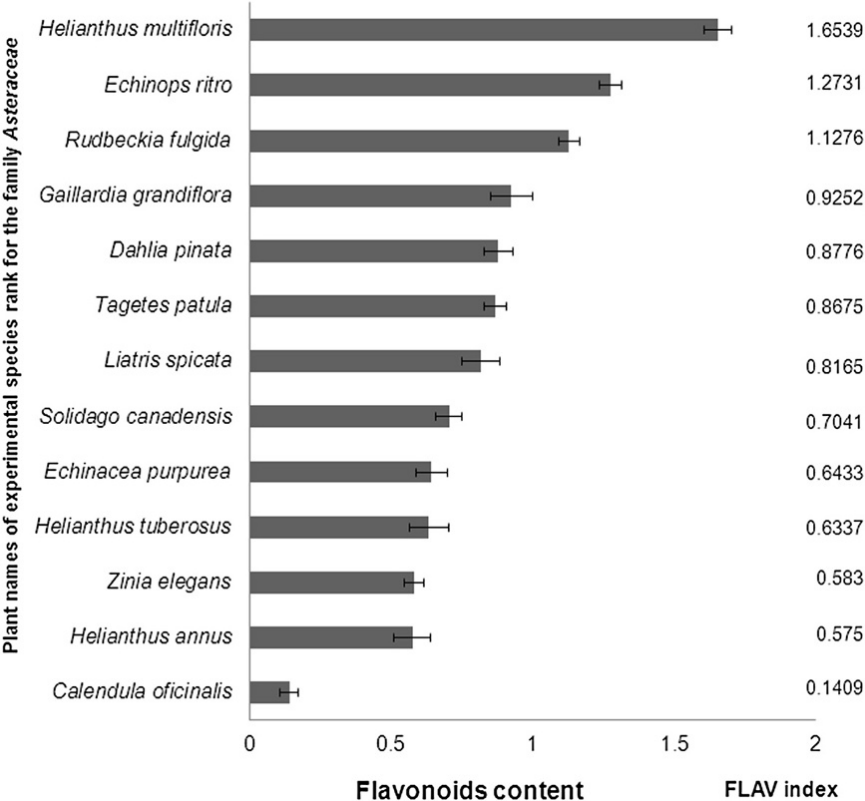
**Content of flavonoids in the leaves of investigated plant species of the family** ***Asteraceae.***

The content of flavonoids has been evaluated in 13 plant species of family *Asteraceae* (Figure 1). Among monitored plants of the family *Asteraceae* have been found the maximum value of flavonoids in the leaves of sunflower (*Helianthus multiflorus* 1.65 RU). Lowest content of flavonoids has been observed in the leaves of marigold (*Calendula officinalis* 0.14 RU). Raal and Kirsipuu [23] address the spectrophotometric determination of the amount of flavonoids in different varieties of *Calendula* species, with the highest amount of flavonoids (0.68%) identified in yellowish inflorescences (Finnish varieties) and the lowest value (0.21%) in orange inflorescences (Estonian varieties). It was concluded that the total content of flavonoids may depend from variety, location and time of growing *Calendula officinalis* seeds; the color of inflorescences does not indicate the total content of flavonoids in the same variety [23].

For another two representatives of *Heliantus* sp. - *Helianthus annus* and *Helianthus tuberosus* content of flavonoids reached only 65% and 62% compared to the content of flavonoids in the leaves of *Helianthus multiflorus*. These results indicate that flavonoid content can be significantly different even in the representatives of one genus. At the same time leaves contain the most allelochemicals because those in roots are lost by leaching and those from stems are translocated [24].

In the leaves of *Echinops ritro* has been found second highest flavonoids content (after *Helianthus multiflorus*) among monitored plants of the family *Asteraceae* (Figure 1). But the content of flavonoids in the leaves of *Echinops ritro* reached 25% which is less compared to the content of flavonoids in the leaves of *Helianthus multiflorus. Echinops* species (*Echinops echinatus*, *Echinops niveus* and *Echinops integrifoliu*s) are known as species with high flavonoids content [25–27].

The high flavonoid content (1.13 RU) have been found in the leaves *Rudbeckia fulgida* (orange coneflower), a species of flowering plant of the family *Asteraceae*, native to eastern North America. The data regarding flavonoid or phenolics content has been mostly presented for *Echinacea purpurea* but not for *Rudbeckia fulgida* (orange coneflower). In the leaves of orange coneflower, the flavonoid content was at two times higher compared to the leaves of purple coneflower (*Echinacea purpurea*).

In the another monitored plant of family *Asteraceae* - purple coneflower (*Echinacea purpurea*) the content of flavonoids in the leaves got value of 0.64 RU. The *Echinacea purpurea* extracted with a 55% ethanol at 55°C contained 86.0 ± 4.6 mg quercetin equivalent g^-1^ of flavonoid content [28]. It is known that the activity of antioxidant and their content (phenolics, flavonoids) of different plants is dependent on extracting solvents such as absolute methanol, ethanol, acetone and ethyl acetate which also makes a difference during estimation of plant antioxidants [29].

In the leaves of French marigold (*Tagetes patula*) has been estomated flavonoid content - 0.87 RU which represents the middle level of flavonoids content among assessed plant species of family *Asteraceae*. Rop et al. [30] investigated the flavonoid content in the flowers of French marigold *Tagetes patula* and found 1.90 kg rutin g^-1^ of fresh weight. The authors concluded that flavonoids synthesis may be conditional on the genetic origins of various kinds of flowers.

At the leaves of *Gaillardia grandiflora*, the blanket flowers, it has been estimated the flavonoid content 0.93 RU which was less on 44% compared to the highest content of flavonoids in the leaves of *Helianthus multiflorus*. Cytotoxic compounds with flavonoid nature from the leaves of *Gaillardia aristata* Pursh. have been established. Ten compounds, neopulchellin, 6α- hydroxyneopulchellin, β-sitosterol-3-*O*-β-D-glucoside, apigenin, quercitin, eupafolin, kaempferol-3-methoxy-7-*O*-α-L-rhamnoside, apigenin-7-*O-β*-D-glucopyranoside, α-amyrin and β-sitosterol were isolated from the leaves of *Gaillardia aristata* by applying bioassay guided fractionation [31]. The contents of flavonoids in the leaves of *Dahlia pinnata* (0.88 RU^),^*Liatris spicata*, dense blazing star (0.82 RU), *Solidago canadensis*, Canada golden-rod (0.70 RU) were on the avarage level among investigated representatives of family *Asteraceae*. The flavonoid pigments of *Liatris spicata* were isolated and identified as the 3-glucoside, 3-rutinoside and 3-glucoside-7-rhamnoside of quercetin [32]. *Solidago canadensis* is typical of a flavonoid-rich herb and flavonol quercetin and its glycosides quercitrin and rutin, found as major constituents of ethanol extracts [33]. Air dried herbs of *Solidago canadensis* were extracted with methanol and HPLC analysis revealed phenolics (chlorogenic acid, caffeic acid, kaempferol-3-O-α-L-rutinoside (nicotiflorin), quercetin-3-O-β-D-rutinoside (rutin), quercetin-3-O-β-D-galactoside (hyperoside), quercetin-3-O-β-D-glucoside (isoquercitrin), quercetin-3-O-β-D-rhamnoside (quercitrin), kaempferol-3-O-α-L-rhamnoside (afzelin) and quercetin from *Solidaginis* herba [34].

**Figure 2:**
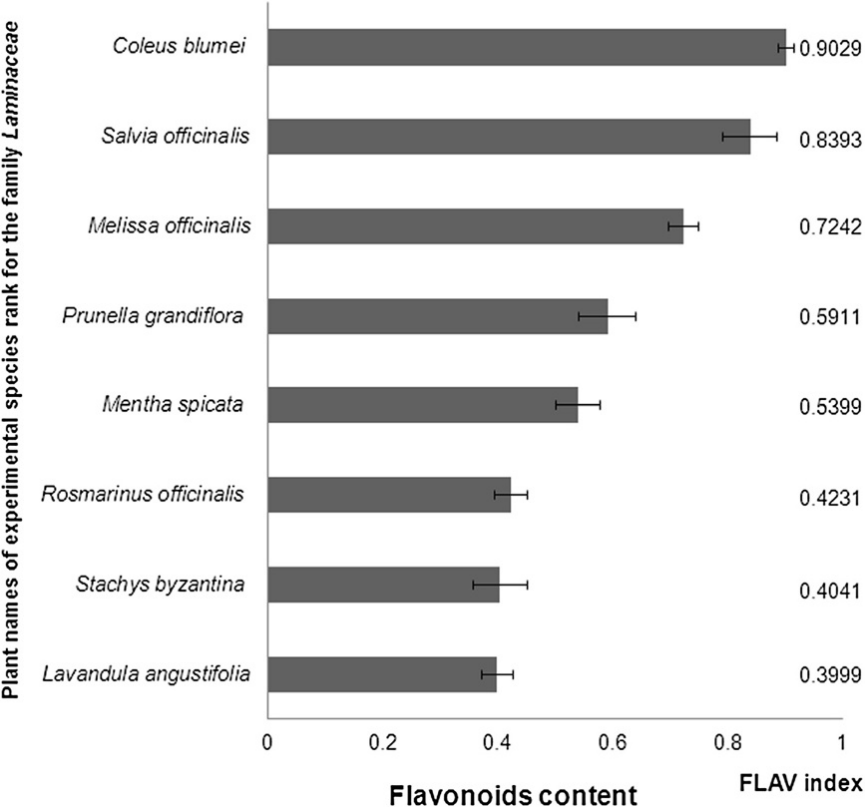
**Content of flavonoids in the leaves of investigated plant species of the family** ***Lamiaceae.***

In the family *Lamiaceae* has been investigated flavonoid content in the 8 medicinal plant species (Figure 2). Range of flavonoids in the monitored species of the family *Lamiaceae* ranged from 0.40 to 0.90 RU. The lowest flavonoid content (0.40 RU) have been detected in the leaves of *Lavandula angustifolia*. In the known herb rosemary (*Rosmarinus officinalis*) has been observed also lowerst flavonoid content (0.42 RU) among investigated plants. The many investigations of flavonoid content were done with air dried herbs extracts of different medicinal plants but these days there are not sufficient data about content of flavonoid content in the leaves or another part of herbs. For example Yoo et al. (2008) with colorimetric determination has been investigated a number of flavonoids in the leaves of 17 selected herbs. The chamomile (*Chamaemelum nobilis* L.), rosehip (*Rosa rubiginosa*), hawthorn (*Crataegus pinnatifida*), lemon verbena (*Aloysia triphylla*), green tea (*Camelia sinensis* L.), black tea (*Camelia sinensis* L.) got highest flavonoid content in the herb extracts among investigated plant species. Among all 17 evaluated herbs the rosemary (*Rosmarinus officinalis*) herb extract got the second highest flavonoid content (448.4 mg catechin 100 g^-1^ fresh weight). In the lavender (*Lavandula angustifolia* Mill) was found mean content flavonoids (390.4 mg catechin 100 g^-1^ fresh weight) [35].

Average value of flavonoids was found in the leaves of lemon balm (*Melissa officinalis*) (0.72 RU). Atanassova et al. [36] identified the highest flavonoid content of lemon balm (45.06 mg catechin 100 g^-1^ dry weight) and in the sage (*Salvia officinalis*) found average amounts of flavonoids (27.54 mg catechin 100 g^-1^ dry weight) among the evaluated herbs [36]. The results of Multiplex measurements recorded in the sage leaves second highest flavonoid content (0.84 RU) after coleus (*Coleus blumei* = syn. *Solenostemon scutellarioides*), where flavonoid content was 0.90 RU. Leaves of sage (*Salvia officinalis*) and *Coleus blumei* with purple leaves were shown to be significantly higher in the flavonoid content compared to the other experimental species of *Lamiaceae*.

*Coleus blumei* (*Lamiaceae*) is an ornamental plant, growing all over the world in an enormous number of different cultivars that vary in color *Coleus blumei* so interesting is the incredible foliage, with arrays of color combinations unmatched by other species and shape of the leaves [37]. *Coleus blumei* has an interesting ability to change its leaf color depending on the intensity of the sunlight [38]. The amount of flavonoid in dried leaves ranged from 0.18 - 15.21 mg QE g^-1^ dried samples. There were significant differences (P *<* 0.05) in flavonoid content among the six *Lamiaceae* leaves extracts. *Coleus blumei* – purple leaves extract contained significantly (*p <* 0.05) higher amount of flavonoid compared to other leaf extracts, while the extract of *Coleus amboinicus* had significantly lowest flavonoid content. Briefly, the highest amounts of flavonoid was in *Coleus blumei* – purple leaves followed by *Coleus blumei* – red leaves, *Coleus amboinicus, Coleus aromaticus* and *Pogostemon cablin* [39].

Family *Rosaceae* is a well-known family for the presence of anticancer, antioxidant compounds. Previously other constituent’s as flavonoids, phenolic acids [40, 41], tannins [42] have been found in this genus.

**Figure 3:**
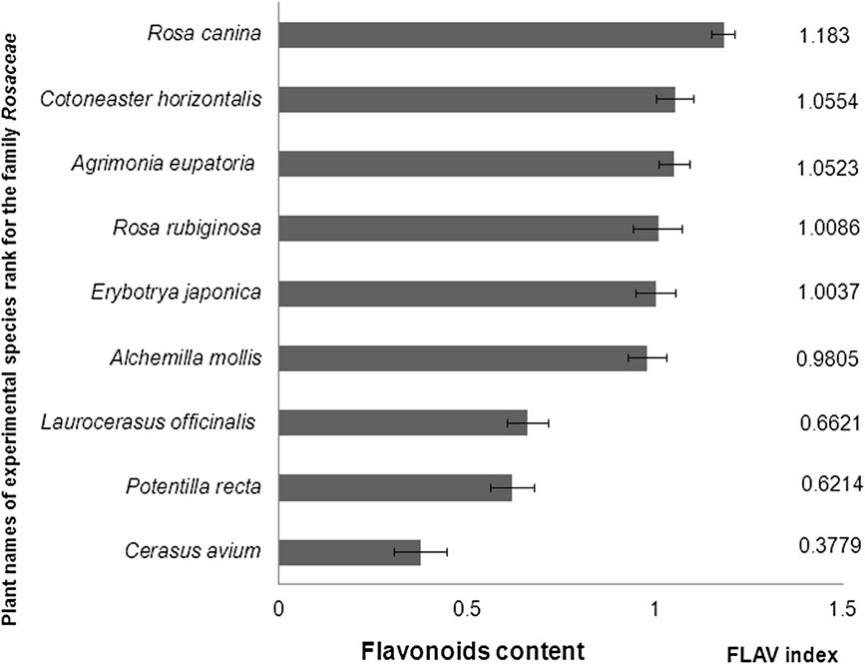
**Content of flavonoids in the leaves of investigated plant species of the family** ***Rosaceae*** **.**

At Figure 3 is shown flavonoid content of 10 monitored species of the family *Rosaceae*. The highest value of flavonoids was determined in the leaves of dog rose (*Rosa canina*) (1.18 RU). Flavonoid content in the family *Rosaceae* has been ranged from 0.38 RU to 1.18 RU and decreased in the order: dog rose (*Rosa canina*) 1.18 RU > cotoneaster (*Cotoneaster horizontalis*) 1.06 RU > agrimony (*Agrimonia eupatoria*) 1.05 RU > rusty rose (*Rosa rubiginosa*) 1.01 RU > japanese loquats (*Eriobotrya japonica*) 1.00 RU > soft lady's mantle (*Alchemilla mollis*) 0.98 RU > laurel medicinal (*Laurocerasus officinalis*) 0.66 RU > direct cinquefoil (*Potentilla recta*) 0.62 RU > bird cherry (*Cerasus avium*) 0.38 RU.

In the leaves of dog rose (*Rosa canina*) was observed the second highest flavonoid content - 1.18 RU (Figure 3). Nowak and Gawlik-Dziki [43] estimated amount of flavonols by HPLC (quercetin and myricetin) in the extracts of the leaves of several species of the genus *Rosa. Rosa canina* showed low flavonol content of 8.53 mg.g^- 1^ dry matter, while *Rosa rubiginosa* showed the second highest value flavonols 18.27 mg.g^-1^ dry matter. The authors suggest that rose’s extracts could be used as natural antioxidants and as part of functional food [43].

The flavonoid content in the leaves of *Cotoneaster horizontalis* has been shown to be the second highest among investigated representatives of family *Rosacea*. Quantitative determination of the total polyphenols and flavonoids of aerial parts of *Cotoneaster horizontalis* Decne family *Rosaceae* was performed colorimetrically with using Folin-Ciocalteu and aluminum trichloride methods. The flavonoid and flavonol contents were expressed as rutin equivalent - 6.8+ 0.76 and 2.2 + 00 mg g-1 respectively. HPLC analysis of total flavonoids showed the presence of three flavonoids (quercetin, naringenin, luteolin) and luteolin was the major compound (9.20 mg/100 g dried plant) [44]. The phytochemical analysis of the ethanolic extract of branches *Cotoneaster horizontalis* revealed the presence of: β-carotene, ascorbic acid, less amount of α-tocopherol and amygdalin (vitamin B17). Information about flavonoids content and composition in the leaves of *Cotoneaster horizontalis* in the literature data is missing nowadays.

Average values of flavonoids have been recorded in the herb rusty rose (*Rosa rubiginosa*) (1.01 RU) and in the leaves of agrimony (*Agrimonia eupatoria* 1.05 RU) (Figure 3). Yoo et al. (2008) has been determined with colorimetric method in the leaves of rose rusty amount of flavonoids 400.5 mg catechin 100 g^-1^ of fresh matter, which belonged to the group with a high content of total flavonoids evaluated herbs [35]. Kubínová et al. [45] has been estimated the highest flavonoid content (3.5 mg quercetin g^-1^ dry weight) in a methanolic extract of the flowering aerial parts of agrimony among evaluated 5 species of the genus *Agrimonia* [45].

The high flavonoid content was estimated in the leaves of *Agrimonia eupatoria. Agrimonia eupatoria* plant is rich in chemical constituents (flavonoids, tannins, aromatic acids, triterpenes, coumarins, terpenoids, glycosides, and vitamins B and K) that can mediate anti-oxidant, anti-bacterial and anti-inflammatory effects [46]. Dried aerial parts of *A. eupatoria* (leaves, stem and flowers) were used for preparation aqueous and methanol extracts and study anti-tumor potential of these extracts. Chemical analysis of *A. eupatoria* extracts (aqueous and methanol) revealed several secondary metabolites. Both aqueous and methanol extracts were positive for flavonoids, alkaloids, tannins and glycosides and negative for saponins. Flavonoids were further identified by thin layer chromatography (TLC), and as suggested by RF values of the separated extracts, the aqueous extract contained myricetin, azoleatin, vitexin and isoorientin, while the methanol extract contained kaempferol, quercetin, isorhamnetin and myricetin [47].

Loquat (*Eriobotrya japonica* Lindl.) is a perennial subtropical fruit tree and application multiplex fluorimetric sensor for analysis of flavonoid content revealed high content of flavonoids in the leaves of loquat. Many studies demonstrated that large amounts of flavonoids and phenolics were found in the fruit and leaf of loquat [48–50], and both the methanol extract of loquat leaf and its individual fraction exhibited strong antioxidant capacity [51]. Methanol had the highest extraction efficiency among five solvents, followed by ethanol. Considering the safety and residue, ethanol is better as extraction solvent. The average content of flavonoids and phenolics of loquat flower of five cultivars were 1.59 ± 0.24 and 7.86 ± 0.87 mg g^-1^ DW, respectively, when using ethanol as extraction solvent. The contents of both bioactive components in flowers at different developmental stages and in the various flower tissues clearly differed, with the highest flavonoids and phenolics content in flowers of stage 3 (flower fully open) and petal, respectively [15]. These data again confirm our suggestion regarding important role of pre-screening with application multiplex fluorimetric sensor with aim to estimate flavonoids content in the different parts of plant which can make more easy choice to select part and species of medicinal herb with high flavonoid content for extraction and further identification flavonoids composition.

Among investigated representatives of family *Rosacea* many of them got higher flavonoid content in the leaves compared to the representative’s familiy *Lamiaceae*. For example in the *Alchemilla mollis* leaves has been found higher flavonoid content (0.98 RU) compared to the *Coleus blumei* and *Salvia oficinalis* (0.84 RU^).^ Different studies showed that the flavonoid compounds present in the plant are responsible for the pharmacological activity of Lady’s mantle [51]. The aerial flowering parts of the plants *Alchemilla mollis* were collected within phenophase – full blossoming, airdried and used for preparation ethanolic extracts. Further purification by RP-18 CC led to the isolation of eight flavonoid glycosides: *cis*and *trans*-tiliroside, rhodiolgin, hyperoside, isoquercitrin, miquelianin, sinocrassoside D2, and gossypetin-3-*O*-b-D-galactopyranosyl-7-*O-*α-L-rhamnopyranoside [52].

This is fact that for phytotherapy needs and research investigation mostly were used aerial flowering parts or aerial parts of plants. But for better knowledge what exist in each part of medical herb (leaves, stems or inflorescences) to use application multiplex fluorimetric sensor for pre-screening flavonoid content is new stage in the area of modern plant physiology and phytotherapy.

It was indicated that, both the water extract of cherry stem (WECS) and ethanol extract of cherry stem (EECS) have antioxidant and antiradical properties, and there is a correlation between these properties and the phenolic and flavonoid contents. Quantities of quercetin, α-tocopherol, pyrogallol, ascorbic acid and other phenolic acids were detected by high performance liquid chromatography and tandem mass spectrometry (LC–MS/MS) [53]. In this experimental work with application of Multiplex fluorimetric sensor for screening flavonoid content in the leaves of different representatives of family *Rosaceae* the leaves of bird cherry (*Cerasus avium* L.) has small flavonoid content. Average flavonoid content have been found in the leaves of laurel medicinal (*Laurocerasus officinalis*) and direct cinquefoil (*Potentilla recta*). But in the leaves of *Laurocerasus officinalis* and *Potentilla recta* flavonoid content was higher than in the leaves of *Cerasus avium* L. on 25%.

## Conclusions

Results of this study indicated that leaves of medical herbs belonging to families *Asteraceae, Laminaceae* and *Rosaceae* can be source of flavonoids, but more detailed biochemical analysis of flavonoids composition is needed. Among investigated plant species, the highest amount of total flavonoids have been found in the leaves *Helianthus multiflorus* (1.65 RU) and *Echinops ritro* (1.27 RU) belonging to family *Asteraceae*, *Rosa canina* of the *Rosaceae* family (1.18 RU) and *Coleus blumei* family *Lamiacea* (0.90 RU). The lowest values were found in the leaves of *Calendula officinalis* (0.14 RU). Our data on interspecific variability in content of UV-absorbing compounds (mainly flavonoids) in the leaves of representatives of medical herbs belonging to families *Asteraceae, Lamiaceae* and *Rosaceae*, confirmed and suggested an important role of pre-screening flavonoid content analysis with application multiplex fluorimetric sensor (MPx). Estimation of flavonoid content in the different plant species and their parts can facilitate selection of species as well as part of medicinal plant with high flavonoid content. Thus, for laborious and expensive procedures, such as extraction and further spectrophotometric, TLC, HPLC, LC–MS/MS identification flavonoids composition can be used the most promising candidates only, which may substantially increase the success of selection. It’s known that activity of antioxidants and their content (phenolics, flavonoids) in the different plants is depend from kind of extracting solvent such as absolute methanol, ethanol, acetone and ethyl acetate which makes difference in the results during estimation of plant antioxidants compared to the results of pre-screening of flavonoids content by MPx fluorimetric sensor. Anyway, we believe that the application of a non-destructive tool, such the MPx sensor, to select top-quality part of plants or medicinal herbs can considerably improve the production of phytoterapeutical products, novel and functional foods. Fluorescence-based sensors can be used directly in the nature or field conditions, with the advantage of taking into account seasonal influences on plant flavonoids, due to the variability of climatic conditions such as rainfall, temperature and irradiance. The MPx sensor could be easily integrated in online sorting devices using the index of flavonoids content as an additional quality parameter for storability too.

## Methods

### Plant material

Determination of flavonoids by application of multi-spectrally induced fluorescence records, we carried out the plants located in the Botanical Garden of Slovak agricultural university in Nitra during flowering period. Flavonoid content was detected in 3 families of medicinal herbs *Rosaceae, Asteraceae* and *Laminacea* with numerous observed plant species. The light-exposed side of leaves (20–25 records in each species) was measured by the fluorimetric sensor [53].

### Multiplex fluorimetric sensor

The Multiplex (MPx) fluorimetric sensor (Force-A, Orsay, France) is described in detail elsewhere [54]. It measures fluorescence emitted by chlorophyll, in the red (RF) and far-red (FRF) spectral regions, under excitation with different light-emitting diode (LED) sources in the UV (375 nm) and visible (blue at 450 nm, green at 515 nm and red at 630 nm). Three synchronized photodiode detectors recorded fluorescent yellow, red and infrared fluorescence [53]. Flavonoid content was determined as the FLAV index, which is derived from UV absorption properties of flavonoids.

The intensity of the chlorophyll fluorescence (ChlF) emitted by a sample depends on the amount of excitation light able to reach the Chl pigment, that is on the transmittance of the epidermis at the excitation wavelength [55]. Flavonols in the epidermis can attenuate part of the incident radiation in the UV-A region before this can reach the Chl molecules. Since the long wavelengths of visible light are not absorbed by flavonols, ChlF excited by green or red wavelengths can be considered as a reference signal. To calculate the relative amount of UV-absorbing compounds, two ChlF signals under UV (FRF-UV) and red excitation (FRF-R) can be used to obtain an index proportional to the flavonoid content in the leaf epidermis [56]:


Measured flavonoid content in the different plant species are expressed in relative units (RU), because the ratio of the optical densities of the two gives a dimensionless number.

### Statistical analysis

The means and standard deviations were calculated by the Microsoft Office Excel 2003. Significant differences of these data were calculated using analysis of variance (ANOVA-Duncan’s multiple test (STATISTICA 10, StatSoft, Tulsa, USA). All results were expressed as mean ± standard deviations from replications n = 20-25.
